# The Search for Reliable Biomarkers of Disease in Multiple Chemical Sensitivity and Other Environmental Intolerances

**DOI:** 10.3390/ijerph8072770

**Published:** 2011-07-01

**Authors:** Chiara De Luca, Desanka Raskovic, Valeria Pacifico, Jeffrey Chung Sheun Thai, Liudmila Korkina

**Affiliations:** 1 Tissue Engineering & Skin Pathophysiology Laboratory and 2nd Dermatology Division, Dermatological Research Institute (IDI IRCCS), Via Monti di Creta 104, Rome 00167, Italy; E-Mails: d.raskovic@idi.it (D.R.); v.pacifico@idi.it (V.P.); l.korkina@idi.it (L.K.); 2 Natural Health Farm, 39 Jalan Pengacara U1/48, Seksyen U1, Temasya Industrial Park, Shah Alam Selangor 40150, Malaysia; E-Mail: jessie@naturalhealthfarm.com

**Keywords:** multiple chemical sensitivity, chronic fatigue, fibromyalgia, electric hypersensitivity, amalgam toxicity, environmental medicine, detoxification, oxidative damage, environmental intolerances, disease biomarkers

## Abstract

Whilst facing a worldwide fast increase of food and environmental allergies, the medical community is also confronted with another inhomogeneous group of environment-associated disabling conditions, including multiple chemical sensitivity (MCS), fibromyalgia, chronic fatigue syndrome, electric hypersensitivity, amalgam disease and others. These share the features of poly-symptomatic multi-organ cutaneous and systemic manifestations, with postulated inherited/acquired impaired metabolism of chemical/physical/nutritional xenobiotics, triggering adverse reactions at exposure levels far below toxicologically-relevant values, often in the absence of clear-cut allergologic and/or immunologic involvement. Due to the lack of proven pathogenic mechanisms generating measurable disease biomarkers, these environmental hypersensitivities are generally ignored by sanitary and social systems, as psychogenic or “medically unexplained symptoms”. The uncontrolled application of diagnostic and treatment protocols not corresponding to acceptable levels of validation, safety, and clinical efficacy, to a steadily increasing number of patients demanding assistance, occurs in many countries in the absence of evidence-based guidelines. Here we revise available information supporting the organic nature of these clinical conditions. Following intense research on gene polymorphisms of phase I/II detoxification enzyme genes, so far statistically inconclusive, epigenetic and metabolic factors are under investigation, in particular free radical/antioxidant homeostasis disturbances. The finding of relevant alterations of catalase, glutathione-transferase and peroxidase detoxifying activities significantly correlating with clinical manifestations of MCS, has recently registered some progress towards the identification of reliable biomarkers of disease onset, progression, and treatment outcomes.

## 1. Introduction

Health concerns for the exponential increase of environmental intolerances, including both allergic and non-allergic sensitivity-related phenomena, is rising in the general public as well as in the medical community [1,2]. Therefore, different environment-associated multi-organ conditions, in most cases still lacking a consensus on clinical case definition, such as multiple chemical sensitivity (MCS), fibromyalgia (FM), chronic fatigue syndrome (CFS), sick building syndrome (SBS), hypersensitivity to electro-magnetic fields (EHS), and others, in most cases still lacking a consensus on clinical case definition, have been subjected in the last decade to clinical and laboratory studies [3]. These are aimed at proving any possible organic cause, or oppositely the psychogenic etiology, as proposed by part of the clinicians on the basis of the prevalent neurologic impairment [3,4]. Main difficulties towards a clinical consensus on disease classification lie in: (i) the wide array of symptoms and signs allegedly linkable to environmental triggers exposure, (ii) the diversity of the subjects affected, reacting on the basis of individual sensitivity and possibly genetic predisposition, (iii) the mere absence of proven pathogenic mechanisms and consequently of clear-cut diagnostic criteria, (iv) the wide spectrum of possible triggers [2] and the absence of clear dose-dependent reactions, generating methodological difficulties and bias in provocation studies. On this basis, the World Health Organization has initially defined this group of invalidating conditions as “idiopathic environmental intolerances” (IEI) [5], with special reference to MCS. Awaiting for adequate mechanism elucidation, these complex and chronic conditions have then also been appointed with a second collective discouraging label of “medically unexplained symptoms” [3,6].

A more appropriate collective definition, implicating acceptable case definition and excluding any preventive negation, appears that of sensitivity-related illnesses (SRI), defined as adverse clinical states elicited by exposure to low-dose diverse environmental-borne physical-chemical triggers [1]. SRI are classified as self-reported or objectively diagnosed aberrant responses of different severity, from mild inflammatory reactions to life-threatening multi-organ impairments [7], in response to a wide spectrum of possible exogenous triggers, through airborne, cutaneous or nutritional exposure at sub-toxic levels, ordinarily not generating any detectable negative effect on the general population [8]. The onset of clinical symptoms connected with SRI, and in particular with MCS, is related to different physical, chemical or biological factors, mainly xenobiotic chemicals, drugs, and metals, electro-magnetic or nuclear radiation, iatrogenic factors including synthetic implants [9], specific foods, microbial and environmental allergens. This cluster of variable conditions is manifested mainly in adult life, with higher prevalence in the female sex [10–12], though reports of pediatric cases see recently an increased frequency [13,14], and evidences are accumulating for a role of *in-utero* sensitization [15]. The initiation of the disease state is commonly self-reported as a single precipitating event of severely intoxicating overexposure, or as a chronic exposure to lower doses of an environmental pollutant, of a kind that may be totally unrelated to subsequent triggering molecules acting during the phase of established disease [1].

There exist conceptual difficulties in attributing a disease *status* to the paradoxical reaction observed in MCS to chemico-physical stimuli delivered in concentrations far below threshold levels established for environmental compounds by the conventional toxicology approach [16–18]. The general assumption of a hormetic (biphasic) behaviour for xenobiotics on biological systems [19] may allow new scenarios, overcoming the threshold dose-response model, and introducing the concept that an environmental toxicant may induce the opposed effects of stimulation/adaptation or toxicity, respectively at very low or high concentrations. The complex network of hormetic response pathways might be altered at some unknown point(s) in MCS subjects, through mechanisms still to be investigated. These theoretical hindrances, back-shielded by protective interests of the industrial and pharmaceutical world, may alone justify the persisting general scarce attention of the public health systems worldwide to self-reported chemical sensitivities, estimated to involve some 10–36% of the civil population, with lower but still very significant figures in the case of clinically diagnosed MCS, (for a timely review, see [20]), leading to partial or complete working and social disability in a relevant percent of cases [21].

The great majority of chronic symptoms referable to SRI are shared by the different so far idiopatic conditions of MCS, FM, CFS, SBS, irritable bowel syndrome (IBS), Persian Gulf War veteran syndrome, amalgam disease, EHS, burn-out syndrome, *etc.* [2,9,22–24]. Notably, in our case history of 620 Italian patients with symptoms referable to MCS, admitted to red-ox marker diagnostic service, we found 35% of cases reporting concomitant hypersensitivity to electromagnetic fields (EMF), approximately 10% of CFS and of FM co-morbidities, and 5% of cases reporting intolerance to multiple dental amalgam fillings (*unpublished data*). Some authors view this as a proof of the functional somatic syndrome hypothesis, where symptoms or diagnostic criteria overlap to indicate a prevailing common psychosocial denominator of the disorders [25]. Conversely, from the biological perspective, the marked similarity of symptoms may also support the expectations for a possible identification of common organic etiological biomarkers of disease. This possible finding does not necessarily imply a clinical overlapping of the different syndromes, which may well represent completely separated clinical settings, sharing some common molecular pathways.

In addition, CFS, FM and the Gulf War syndrome show specific features, which are co-morbid with known autoimmune diseases like systemic lupus erythematosus [26], rheumatoid arthritis [27], or vitiligo [18]. Indeed, the question as to whether SRI should be considered diseases or plain clusters of symptoms remains unanswered by available conventional clinical interpretations of acute/chronic inflammatory processes [3,28], since patients only partly display recognized dysfunctions of the immune system, or IgE-based allergic reactions, or any detectable anatomic or functional organ alteration unequivocally connected with trigger challenge.

The need to address these issues systematically, employing experimental research as well as clinical tools, to provide growing patient complaints with adequate responses, still remains largely unmet [1,29,30]. As a main consequence, the absence of recognized diagnostic and therapeutic guidelines represents a compelling issue, leaving space for the application of non-standardized and non-validated protocols. The lack of satisfactory reliability and specificity of the biomarkers presently measured in the clinical setting, and the almost absolute void of epidemiological data on treatment safety, efficacy and compliance assessment by the responsible clinicians, raise undeniable ethical concerns for public health operators [31] and for regulatory agencies in the civil and professional domains [32].

Here, we provide an overview of the main groups of environmental hypersensitivities, and of the state-of-the art of research on possible specific biomarkers of disease, in support of a comprehensive case definition of multiple chemical sensitivity and other related conditions as organic pathologies. The consequences of the scarce knowledge of the possible etiology of the diverse conditions on current therapeutic approaches and on sanitary and social public awareness are highlighted, supporting the urgent need for a renovated and more intense experimental and epidemiologic research. Directing efforts towards the individuation of specific biomarkers of environmental hypersensitivity may also prove diagnostically useful, and bring new mechanistic insights into other recognized, difficult-to-cure, chronic inflammatory conditions with suspected environmental-borne etiological co-factors, like atopic or autoimmune skin diseases, as regards prognostic or therapeutic indicators, and nutritional/lifestyle recommendations.

## 2. Controversial Case Definitions

### 2.1. Multiple Chemical Sensitivity

Multiple chemical sensitivity is described as an acquired multi-organ condition, where recurring symptoms are muscular weakness and fatigue, confusion and memory loss, minor and major depression, general anxiety, panic disorders and post-traumatic distress, respiratory distress, chronic bronchitis and asthma, ear-nose-throat disturbances, autoimmune disorders, gastrointestinal and genitourinary tract malfunction, and migratory joint pains [23,33]. Prevalence studies are complicated by the uncertain diagnostic criteria, nevertheless epidemiological studies have estimated up to 15% of the USA population in the scholar and working age to be affected with chemical sensitivity at varying severity degrees [34]. Events of exposure to trace concentrations of common odorous substances, including volatile organic compounds (VOCs), perfumes, fresh paint, cleaning chemicals, print and toners, carpeting, and numerous other products, are self-reported as connected to the onset and perpetuating of symptoms by MCS patients, although it is widely stated that adverse reaction is provoked as well by inodorous incitants, primarily selected drug categories and water or food additives and contaminants [35]. A relevant role is also attributed to airborne or food microbial load, especially mould contamination, heavily called into play also for sick building/house syndromes, a frequent comorbidity in MCS [36].

The diagnosis is set on the basis of anamnestic criteria and thorough enquiry on possible trigger exposure, through the medically assisted compilation of validated questionnaires [8,37]. Due to the prevalence of neurologic symptoms, and to the persisting lack of recognized molecular markers for MCS, clinicians incline to classify it among the somatoform disorders, on the basis of several studies demonstrating unsatisfactory results of provocation studies, thoroughly reviewed by Das-Munshi *et al*. [38]. The rather numerous and diverse leading pathogenetic hypotheses postulated for this syndrome take into consideration both organic and psychiatric/psychological factors. Among the possible somatic mechanisms, the more developed approaches include immunological dysregulation, neurogenic inflammation, limbic kindling and neural sensitisation, toxicant-induced loss of tolerance, altered xenobiotic metabolism, altered nitric oxide/peroxynitrite cycle, behavioural conditioning, psychological/psychiatric factors (for recent extensive review, see [20]).

A relevant part of the clinical and experimental data addressing organic/molecular causes accounts for an impairment of the chemical defensive system as a suitable mechanism underlying the MCS condition. Indeed, adaptation to the chronic non-physiological load of conventional compounds, such as cosmetics, detergents, preserving agents and eccipients, pharmaceutical drugs, as well as to brand new molecules, like slow degrading nanomaterials for medicinal use [39] characterizing the rapidly changing environment, requires an extraordinary metabolic effort. The concerted action of constitutive and inducible, strictly regulated, protective pathways, featuring this metabolic chemical-defensive network, has presumably evolved very early in primitive eucariots, far before immune system development, in order to detoxify low-molecular-weight organic and inorganic compounds, including heavy metals, as well as endogenous non-protein signaling molecules, mediators of inflammation, degradation products, and toxic by-product of cellular metabolism [40]. Human complex detoxification system is a common target of inherited or acquired genetic defects and epigenetic factors. Subjects with gene single nucleotide mutations (snips) or deletions display incomplete detoxification of exogenous/endogenous toxins or/and excessive generation of toxic by-products, whilst hyper-functional genes (duplicated or multiplicated) determine higher-than-normal rates of metabolization [41]. Notably, evidences on the possible role of malfunctioning phase I metabolizing enzymes in the susceptibility to non-tumor environment-associated pathologies are still limited and contradictory.

The scant experimental evidence gathered so far in controlled clinical/laboratory studies on MCS patients still poorly support the whole body of medical hypotheses. In particular, available clinical data prove, though only indirectly, that functional or/and genetic defects of endogenous enzymes detoxifying hydrogen-/lipid-peroxides or stable toxic products of lipid peroxidation, may cause chronic oxidative stress and consequent metabolic and immunologic alterations characteristic for the patients with environmental SRI. Our group has recently demonstrated [17] relevant alterations *vs.* healthy controls of erythrocyte catalase, glutathione peroxidase (Gpx), glutathione transferase (GST) activities, and of decreased levels of glutathione and polyunsaturated fatty acids, in association with specific alteration patterns of pro-inflammatory cytokines, significantly correlated with clinical manifestations in a representative group of MCS Italian patients, has registered some progress towards the identification of reliable markers of disease onset and progression. These and other possible validated markers may also be useful for a correct and evidence-based evaluation of treatment outcomes and follow-up, to-date unfortunately still unaccomplished.

### 2.2. Other SRI/IEI

*Gulf War veterans syndrome* is a paradigmatic as well as a paradoxical example for all professional and civil intoxication-borne multi-organ and multi-symptom syndromes. Following the Gulf War of 1991, a series of studies on veterans have documented severe health adverse outcomes, mostly chronic in nature, with a wide variety of clinical signs. Most frequent manifestations included sleep disorders, chronic fatigue syndrome, headaches, gastrointestinal disorders, fibromyalgia, multiple chemical sensitivity, anxiety and depression, sexual disability, post-traumatic syndrome, and more, resulting in a degree of disability not found in military personnel not deployed to the Gulf War [23,42]. Relevant studies, thoroughly reviewed by Thomas *et al.*, [43] have linked these conditions to exceptionally severe and prolonged intoxicating events, most notably multiple vaccinations, exposure to depleted uranium and powerful chemical toxicants present in war theatres. Other conditions connected to this polymorphic disorder are the *post-traumatic stress disorder* [44] and the *burn-out syndrome* [21], displaying similar impairment of the immunologic and neuro-endocrine patterns, and overlapping symptoms.

*Fibromyalgia* is a chronic pain syndrome, often leading to working and social inhability. It is characterized by widespread inflammatory rheumatic disease with non-articular musculoskeletal pain, acute febrile illness, paresthesia, fatigue, primary sleep disorders, memory and concentration impairment [45], major depressive disorder, migraine, and irritable bowel syndrome [46], as stated by consensus diagnostic guidelines [45]. The unavailability of specific anatomic, histological, or molecular disease markers complicates the diagnosis. Environmental factors, including certain foods and chemicals, may trigger the development of the chronic pain disorders in individuals with a genetic predisposition [47] FM is often co-morbid with *Chronic Fatigue Syndrome*, which is diagnosed on the basis of unexplained disabling fatigue lasting for at least six months and not healing with rest, along with several non-specific accompanying symptoms, including disordered sleep physiology and non-restorative sleep syndrome, diffuse myalgia, cognitive and behavioural impairement [48]. The still unclear patho-physiological mechanism bases upon immunological findings, related to altered cytokines profiles, decreased function of natural killer cells, presence of autoantibodies, reduced response of T cells to mitogen and other specific antigens [49]. The activation of peripheral and central inflammatory and oxidative stress pathways has been recently called into play [50].

Chronic intoxication with inorganic mercury due to amalgam fillings is claimed as a major trigger and incitant in MCS [51] and CFS [52]. *Amalgam disease* is as yet a controversial disabling condition, occurring in allegedly metal-sensitive subjects who carry dental fillings, which contain almost 50% mercury amalgam, with tin-copper-silver and zinc. This material, used since almost two centuries in dental care and thus considered safely bio-compatible, release Hg vapors contaminating saliva, though in daily doses far lower than those known to induce neurological symptoms [53]. Indeed, tissue contamination through dental root and jaw bone transmission has also been proven [54,55]. Mercury vapor is biologically oxidized to Hg bivalent cation, the toxic form of mercury deriving from cellular metabolic oxidation, a far more damaging species than lead and cadmium bivalent ions, because of its higher affinity with thiols, causing irreversible inhibition of cystein containing proteins. Inorganic Hg contained in dental amalgam is transformed into organic methyl-mercury by residential microorganisms in the mouth and in the gastrointestinal tract [56,57].

Chronic Hg intoxication has been connected with a wide variety of symptoms, ranging from cutaneous and oral mucosa signs [58] with increased infection susceptibility, to persistent fatigue with joint and muscle pain, neurological symptoms [59] and vegetative disorders, headache, migraine, lack of concentration, low memory capacity, depression, sleeping disorders. Clinical reports have linked dental amalgam implants with prevailing skin and mucosa manifestations of autoimmune diseases [60–62]. Few rigorous evidences have also linked dental amalgam to increased risk of multiple sclerosis, the association with Alzheimer’s or Parkinson’s disease also being investigated [63,64]. Studies on the dentist category have generally documented neither abnormal death and disease rates, nor any disability susceptible of association with Hg vapors, based on the assumption that their urinary or blood Hg levels were not significantly elevated. In particular, no consistent associations have ever been found between urinary Hg concentration, or the chronic index of Hg exposure, and any category of neurological or psychogenic symptoms [65]. It must be noted though, that Hg concentration in body tissues was proven to be independent of urinary, blood, saliva and hair concentrations [66]. Mercury disposition occurs in fact mainly through stools, in a ratio of 12 to 1 to urine excretion, this latter probably reflecting only kidney, and not total-body burden [67,68]. In support, urine levels of Hg below 5 μg/L (the No Observed Adverse Effect Level—NOAEL—for mercury) have been reported in cases of occupational intoxication [69]. Moreover, data assessing a very long-term persistence of elevated Hg tissue levels after chronic or acute professional intoxications are available [70]. Up-to-date, the invalidating complex of symptoms labeled as amalgam disease stays almost unrecognized by both clinical community and legal agencies. Methodological flaws of published studies and possible conflict of interest of dentist professional associations in official position papers have been recently thoroughly reviewed by Mutter *et al.* [71].

*Electro-magnetic hypersensitivity* is a condition perceived to be consequent to the exposure to environmental electro-magnetic stressors, followed by recovery through the complete isolation from triggers [72,73]. Electro-magnetic fields represent indeed one of the major indoor pollutants in the western world. Subjects complain functional symptoms of the nervous system, *i.e.*, dizziness, fatigue, headache, difficulties in concentration, memory problems, anxiety, depression, respiratory, gastrointestinal, eye and vision manifestations, palpitations, chronic fatigue, fibromyalgia, skin inflammatory and allergic reactions of face, hands and forearms. These and other disorders are generally self-reported by patients, in the absence of somatic pathological signs supporting a specific case definition, with the main exception of skin manifestations. Pioneering reports from Scandinavia deal in fact with dermatological symptoms, subjective-itching, burning- and objective-redness, dryness-appearing during work with video display units [74,75]. This syndrome is subjectively attributed to the exposure to frequencies in the radio, microwave, kilohertz, and extremely low-frequency ranges of EMF or radiofrequencies, including the “dirty electricity” due to poor isolation of electric wires and telephonic lines, wireless devices, wi-fi, *etc.*, though no conclusive demonstration of causal relationship has been drawn so far. Physical parameters characterizing these EMF sources vary considerably, therefore irradiation levels are difficult to classify and standardize for provocation studies purposes [76]. Again, in most cases, exposure levels called into play are far below those known to cause physiological changes in animal models [77], or adverse health consequences in industrial settings. Electric pollution has been connected, in limited studies encouraging deeper investigation, to increased risk of chronic and degenerating diseases, including asthma, diabetes, and multiple sclerosis [78].

As in the case of Hg intoxication, compelling evidences are available only on acute and chronic health disorders deriving from professional exposures to high intensity fields, in electric power plants, whilst meager data are available on chronic sub-acute exposures below recommended reference levels, occurring in the civil life. No entirely convincing hypothesis on mode of action has been proposed and proven so far, while provocation studies have provided uncertain results. Data about specific heavy metal blood concentrations of cadmium, mercury and lead in EHS subjects [79] appear relevant, especially in the frame of the considerable overlapping of symptoms with amalgam disease, as related to chronic fatigue, headaches, general malaise and insomnia, although intoxication levels reported are below toxic concentrations. Also in this case, methodological doubts concerning biological samples analyzed, not necessarily representative of effective tissue and organ loads [80], raise the alarm that even negligible metal concentrations may be actively mobilized from tissue under EMF exposure, and exert immuno-toxic effects, with inflammatory cytokine hyper-production and release.

*Irritable bowel syndrome (IBS)* is a rather common gastrointestinal functional disorder characterized by recurrent abdominal pain or malaise due to enteric and central nervous system modified functions, with stool abnormal frequency or consistency, microbial flora abnormalities with altered host-microbiota interactions and immune activation [81], and consequent disturbed defecation, once again in the absence of structural abnormalities and reliable biomarkers of disease. This condition, estimated to have 5–25% prevalence in the world population, again more frequently reported for the female gender, is not presently clarified in terms of patho-physiological mechanism, and is diagnosed exclusively according to symptom classification-based guidelines and exclusion of other known causes of organic pathologies, such as celiac or colon cancer diseases [82]. IBS is a heterogeneous condition presenting very frequent co-morbidity with other disturbances, mainly migraine headache, fibromyalgia and chronic pelvic pain, anxiety and depression, somatization, stress sensitivity, with consequent low quality-of-life scores bearing relevant socioeconomic impact [83]. As in the case of other SRI, investigations about genetic predisposition have led up-to-now to no definite results, in spite of the wide array of candidate genes proposed [84].

## 3. State-of-the-Art of Diagnosis for Environmental Intolerances

### 3.1. Clinical Criteria

At present, specific diagnostic tools are extremely limited and generally not validated, and the whole clinical process suffers from the lack of case definition in the majority of these conditions, except fibromyalgia, diagnosed on the basis of consensus guidelines [85,86]. It is also generally recognized that the identification of all possible agents leading to xenobiotic intolerance in SRI is difficult, due to the wide variety of potential environmental and nutritional triggers and incitants, often very different between the first precipitating event and the following crises [1]. MCS diagnosis is based on the compliance to Cullen’s inclusion anamnestic criteria coupled with exclusion of any other known organic cause [8], and on the scoring resulting from the Environmental Exposure and Sensitivity Inventory (EESI), a standardized instrument for measuring self-reported chemical sensitivities based on a multistep questionnaire [87], or its locally modified forms [88,89].

### 3.2. Diagnostic Testing-Molecular Biomarkers of Disease

The search for reliable, sensitive, and specific organic biomarkers of disease, possibly measurable by non-invasive techniques, remains today the primary unmet urgent goal for SRI diagnosis, and in particular for MCS. The wide variety of proposed etio-pathogenic mechanisms, calling into play neuro-endocrine, immunologic, genetic or metabolic factors, has generated a disparate array of laboratory tests of claimed diagnostic value and specificity, ranging from the determination of a complete array of toxins, organic and inorganic contaminants in different biological samples, immunologic monitoring, genetic tests, oxidative stress markers, *etc.* Neurologic imaging approaches to prove functional brain impairment have also been taken into consideration, due to the prevalence of CNS symptoms. None of the main directions in MCS diagnostics has found till today acceptable levels of consensus within the bio-medical community and the sanitary regulatory organs. We will here limit the discussion to the approaches that are presently most developed.

#### Genetic markers

Advances in toxicogenomics have highlighted the role of inherited genetic traits in the individual susceptibility to both xenobiotics and toxic endogenous metabolites [90]. Evolutionarily, a complex array of gene families coding for enzymes of specific molecular pathways has specialized to detoxify and eliminate toxicants, and to repair molecular consequences of chemical damage. Chemicals entering into cells are subjected to biotransformation by oxidative phase I enzymes in the cytoplasm, primarily by cytochrome 450 enzymes-CYPs-flavoprotein monooxygenase, amine oxidases, xanthine oxidase, frequently followed by phase II reductive or conjugative modification through glutathione-*S*-transferases (GSTs), UDP-glucoronosyl transferases (UGTs), catechol-*O*-methyl transferases (COMT), *N*-acetyl transferases (NATs), epoxide hydrolase, and others [91].

A main function in the transcription of numerous detoxification genes coding for phase I and II metabolizing enzymes, *in primis* the CYP 1 subfamily, Nrf2, and GST, is played by aryl hydrocarbon receptor (AhR) transcription factor, also involved in modulating the expression of a number of pro-inflammatory mediators, such as cytokines and growth factors [92–94]. Reactive oxygen species (ROS) are active part of the detoxification process both in exposed epithelia and in internal tissues, with the double role of biosensors of environmental/endogenous stressors with signaling function, and of by-products of toxicant-metabolizing pathways and toxicant-induced oxidative damage [40,95–97]. Their tissue concentrations are tightly regulated by a complex red-ox homeostatic control. ROS generated as by-products of phase I reactions are rapidly reduced to non-toxic “physiological” levels by antioxidant enzymes, superoxide dismutase, catalase, glutathione peroxidase, peroxiredoxins, and by low-molecular-weight antioxidants, such as reduced glutathione, uric acid, ascorbic acid, ceruloplasmin, lipophilic antioxidants, *etc.* Overexposure to exogenous or endogenous toxicants, through the interaction with cellular membrane or nuclear receptors, induces the expression of an array of stress-responsive genes, coding for bio-sensoring, signal transmission, and response elements. Superoxide anion-radical, hydrogen peroxide, lipid peroxides, and other ROS come here into play as mediators of the activation of protein kinase (PKs) cascades, and/or transcription factors (nuclear factor κB (NFκB), activator protein-1 (AP-1), and antioxidant response element (ARE)-binding proteins), to start gene transcription and protein synthesis of the phase I, phase II, and antioxidant enzymes [40,96,97], and to modulate cytokine-mediated inflammatory response.

Based on the main hypothesis of impaired detoxification system, leading studies on chemically hypersensitive populations have been so far concentrated mainly on the genetic approach (Table 1), demonstrating an increased risk for MCS in Caucasian females homozygous for the CYP2D6 isoform and for the association of CYP2D6 and phase II NAT-2 polymorphisms [98]. Proneness to MCS was connected with NAT-2 polymorphism and homozygous deletions of M1 and T1 GST genes [99]. The poor metabolizing genotype with polymorphism CYP2D6, a key enzyme in the metabolism of most psychotropic drugs, was also proposed as a negative prognostic factor in the development of FM [100]. Of utmost interest for clinical application, GST isoforms over-expression has been strongly linked with an early onset of various diseases based on impaired carcinogen detoxification, since GST polymorphisms may reduce glutathione conjugation, one of the major protective mechanisms to modulate reactive metabolite-induced oxidative damage, particularly genotoxic [101]. Polymorphisms of GST single isoenzymes and combinations contribute to resistance to carcinogens, antitumor drugs, environmental pollutants, and products of oxidative stress, and therefore have been correlated with MCS [99] and FM [102]. The prevalence of neurological symptoms and neuro-muscular pain in MCS, FM, and CFS has also directed investigations towards COMT polymorphisms, since COMT genetic and epigenetic factors are implicated in the impairment of catecholamine regulation, of cognitive tasks, and in the disregulation of nociceptive signalling of NF-kB [103]. Prevalence of the more active COMT allele has been related to panic disorder, in particular in female patients [85], whilst low COMT activity has been associated with increased risk of chronic pain syndromes [47]. UGT polymorphisms in chemical and nutritional intolerances await deeper investigation, since UGT isoforms catalyze the conjugation of different xenobiotics and endobiotics, including drugs, chemical toxicants, carcinogens, phytochemicals, steroids, bilirubin, bile acids, fatty acids, prostaglandins, and most interestingly thyroid hormones, also modulating three UGT isoforms. An impairment in hormone metabolism is particularly suggestive, due to the prevalent onset of MCS in females in the menopausal period [12], and the common finding of thyroid hormone dysfunctions, including autoimmune thyroiditis (our unpublished data) and increased levels of the stress-sensitive thyroxin hormone [104]. These may account for anxiety, depression, or poor quality of life scores, although some studies have denied a role for thyroid hormones in IEI [33,105].

Of particular interest for MCS, both cytosolic GSTs and UGTs are highly expressed in the olfactory epithelium. Concerning other possible malfunctions of the detoxifying enzymes in SRI, the genetic or acquired alterations of peroxide metabolism deserve further investigation, in view of the growing amount of data available on peroxides hyper-production in different environmental intolerances [106,107]. Catalase, selenium-dependent glutathione peroxidases, and thioredoxin-dependent peroxidases are crucial enzymes in the protection from oxidative damage generated by hydrogen peroxide and lipid peroxides. Complete acatalasemia bears severely adverse health outcomes, including diabetes mellitus; lower-than-normal catalase activity implies a high risk of the premature onset of age-related degenerative diseases [108], and has been proven to be a key metabolic feature in MCS patients [17].

As a whole, these studies provide evidence that multiple polymorphisms of drug metabolizing enzymes predispose MCS individuals to exaggerated chemical sensitivity. Nevertheless, other recent works exclude any clear-cut genetic basis for MCS [112]. A three year experience with environmental hypersensitivities has provided our diagnostic centre with a case history today counting 620 Italian patients, with marked prevalence of the female sex (80.6% *vs.* 19.4 % of males), either self-reported or fully diagnosed for MCS, partly with concomitant EHS and/or FCS/FM symptoms. As previously published [17], we have confirmed no significant prevalence of CYP isoforms, UGT or GST gene variants in a selected group of 144 Italian MCS patients *vs.* 218 healthy controls, although erythrocyte GST activity levels were found severely impaired.

Hence, up-to-now studies on Phase I/II enzyme genetic or post-genetic impairment in MCS European and North American populations bear limited and contradictory conclusions, mainly due to inhomogeneous and insufficient size of the patient and control groups, and to different and not standardised patient cohort inclusion criteria adopted by the different studies. Table 1 lists the main gene variants analysed till now in MCS and other SRI.

Independently of any possible etio-patogenetic relevance, the identification of genetic and phenotypic determinants of drug intolerance, which is diagnosed in the routinely clinical practice only on the basis of anamnestic data and skin tests, is of particular practical importance in patients with environmental SRI, where a great number of medications is commonly administered due to multi-organ pathological manifestations. Genetic information enables targeted avoidance of specific molecules aimed at preventing the frequent occurrence of adverse adverse skin or systemic reactions to drugs or nutritional compounds. These reactions are known to occur in female patients with 1.5- to 1.7-fold greater risk, as compared with males [7]. This may at least in part be due to the relatively higher bioaccumulation of xenobiotics coming from the exposure to everyday toxicants contained in cosmetics, soaps, drugs, *etc.*, often starting early in life, and rendering females more predisposed to SRI [1,113]. In fact, the degree of hypersensitivity often parallels the intensity of the total body burden of bio-accumulated toxicants [114]. Gender immunological differences in T cell activation and proliferation, are probably also a base for the observed increased prevalence of systemic lupus erythematosus and photosensitivity in females [7]. In summary, the relevance of extensive genotyping for the clinical management of the patient suffering from multiple sensitivities is doubtless, notwithstanding the elevated costs for both patients and sanitary systems, and the very limited availability of reliable analytical facilities.

As a general rule, the consequences of genetic or functional alterations of detoxifying enzymes require thorough clinical interpretation, as they may be either detrimental or beneficial, depending on the biological function and relative toxicity of the parent compound and of its metabolites. In the case of MCS, clinical interpretation may be further complicated by the presence of multiple gene classes, and by an elevated genetic heterogeneity of the patient population [112], suggesting that the analyzed variants in the genes examined are of less importance to MCS than possible gene-environment interactions or other still unknown determinants [44,67,112]. Among these latter factors, epigenetic modulation of gene expression, involving developmental and environmental pre-birth or post-birth multi-organ influences, promises to offer relevant clues for individualized medicine, in terms of pathogenesis but also of possible new drug development [115,116].

#### Immunologic markers

Cytokine profiles have been analysed in different conditions sharing a chronic inflammatory state, and involving clinical manifestations such as fatigue, fever, sleep disorders, pain, stress, and persistent aching. Specific patterns of deregulation have been found especially in FCS and FM, with increased spontaneous *ex-vivo* leucocyte release [117,118], or impaired TNF-α, IL-6, and heat shock protein release following maximal cycling exercise, positively correlated with abnormally increased TBARS plasmatic levels [119]. Our group has analysed the profile of 27 cytokines, chemokines and growth factors in the plasma of 77 MCS patients, detecting significant elevation of IFN-gamma, IL-10, IL-8, MCP-1, VEGF and PDGF *vs.* a control group of 52 healthy age- and sex-matched subjects [17]. More extensive cytokinomic investigations are definitely needed, also in connection with red-ox homeostasis alterations and oxidative damage to protein targets, which may well trigger immune depression [120] as well as autoimmune phenomena. Functional and biochemical overlapping of SRI manifestations with *systemic lupus erythematosus* and other autoimmune diseases further support the feasibility of an immunologic approach, although currently results for MCS are largely controversial [17,18]. The diagnostic protocols for MCS usually include immunometric tests, firstly autoantibody research. In addition, lymphocyte transformation test (LTT) or its adaptations (MELISA-Memory Lymphocyte ImmunoStimulation Assay) are employed in many private diagnostic centres Europe-wide to detect a specific metal immunotoxicity, inducing delayed type T cell hypersensitivity [121]. So far, the validity of LTT has been demonstrated conclusively only for beryllium hyper-sensitization, but still requires further confirmation in the case of Hg or other common environmental metals [122], frequently called into play in MCS and in several autoimmune diseases, such as autoimmune thyroiditis, lichen, psoriasis, systemic lupus erythematosus and atopic eczema [123]. In addition, a complete allergologic screening with both epicutaneous and blood testing, must in any case always be performed, although inconclusive for MCS differential diagnosis, to exclude concomitant IgE-mediated components.

#### Metabolic markers

In this area, the role of oxidative stress and inflammatory mediators represents a leading direction for future developments. Availability of data concerning the involvement of chronic oxidative and nitrosative damage in the induction and perpetuating of symptoms in different environmental hyper-sensitivity syndromes has been growing in the last decade (for detailed review, see [3,20,124]). Abnormal oxidative stress levels were first reported in FCS patients by Jammes *et al.* [125], demonstrating increased TBARS levels and ascorbic acid consumption in response to incremental exercise, and by Kennedy *et al.* [126], reporting high plasma isoprostane and oxidised LDL levels in obese and nonobese FCS patients. A rather extended list of red-ox imbalance markers has been drawn for both CFS and FM studies, though respective alteration profiles seem to differ sensibly. Muscle tissue oxidative damage to DNA and lipids, elevated blood levels of malonyl dialdehyde and other lipoperoxidation markers, of F2-isoprostanes and protein carbonyls, reduced levels of α-tocopherol (vitamin E), of reduced glutathione (GSH) and its precursors, of total antioxidant capacity, and mild Mg deficiency are common findings. Significant increase *vs.* control of erythrocyte of total and reduced GSH levels, in association with elevated plasma 4-hydroxynonenal (4-HNE), a genotoxic product of omega 6 polyunsaturated fatty acid (PUFA) peroxidation, and with significant decrease of cell membrane PUFA, were also demonstrated in fully diagnosed MCS patients [17]. Concerning metal ion toxicity, often called into play in these syndromes, scarce preliminary information is available on sensitive oxidation-related metabolic biomarkers, *i.e.*, the increased levels of methemoglobin formation in FCS erythrocytes, consistent with the observed impairment of its physiological reducing agents GSH and cystein [3]. The reduced total antioxidant activity in the saliva of healthy subjects bearing multiple amalgam fillings [127] has been putatively attributed to Hg release, although this is questioned by the finding of similarly increased levels of lymphocyte DNA damage in healthy carriers of both amalgam and methacrylate dental restorations [128]. Further basis for a role of inflammatory response system and oxidative/nitrosative damage in dysfunctions like FCS is offered by the significant induction of spontaneous and stimulated NFkB, cyclo-oxygenase, and inducible NO synthase in peripheral blood lymphocytes *vs.* matched controls [129]. In FM patients, markedly increased spontaneous hydrogen peroxide release from circulating granulocytes and elevated plasma levels of oxidation markers were reported, negatively correlated with depression scales [130,131].

According to the hypothesis proposed by Pall [132], chronic peroxynitrite hyperproduction is implicated as a common etiologic factor for CFS, MCS, FM, and post-traumatic stress disorder. Elevated peroxynitrite levels may cause mitochondrial dysfunction, lipid peroxidation and, by positive feedback, elevated pro-inflammatory cytokine levels, in a vicious cycle inducing NO and superoxide production, with additional peroxynitrite formation. The theory is not conflicting with other mechanisms proposed for MCS, namely neural sensitation, neurogenic inflammation and porphyrin pathway aberrations [124], although *in vivo* studies have not as yet unequivocally demonstrated in FM, CFS or other environmental intolerances the supposed increased NO levels, which has been only indirectly confirmed by our group in MCS patients, showing a significant elevation of plasma nitrites/nitrates levels [17].

There again, interpretation of unbalances in red-ox defenses is complicated, in that the up-regulation of detoxifying enzyme activities and low-molecular weight antioxidant levels may be a sign of positive functional adaptation, though at the same time indirectly revealing the occurrence of a highly oxidative *status*. In this connection for example, ubiquinol tissue and blood levels, as well as isoprostane urinary excretion, were recently found similar or even higher, in FM group *vs.* controls [77,133], and up-regulated GST activity was demonstrated in FCS muscle biopsies [134]. To add further reasons to the difficulty of interpretation, data on oxidative and nitrosative stress have been thus far only partly correlated with specific clinical symptoms, and confounding overlapping risk factors, such as obesity, hypertension, smoking, are not always taken into due consideration [126]. As a whole, before being considered for a possible diagnostic value in the cluster of environmental hypersensitivity conditions, these and other proposed biomarkers need to pass a first step for the evaluation of clinical reliability, *i.e.*, the correlation between marker alteration and severity of clinical manifestations, to be confirmed in pilot clinical/epidemiological studies, and for the cost/benefit analysis. A second crucial phase implicates the technical validation of the analytical method for the clinical use, that is the compliance to accuracy, sensitivity, specificity parameters, and a thorough standardization of the analytical procedure [69,135], to be followed by clinical application on the large scale and subsequent outcome evaluation. This process of technological transfer from bench to bedside has been already completely accomplished only for genomic analysis of phase I/II enzyme polymorphisms, more and more routinely applied in oncology and cardiology, to preview individual response to drugs [136], but awaits clinical development for many lipidomic, metabolomic and proteomic/cytokinomic markers, which are expected to contribute substantially to the establishment of an evidence-based personalised medicine.

### 3.3. Diagnostic Testing-Functional Brain Imaging

In the field of physical diagnostics, functional brain imaging has gained some clinical consensus, due to the heavy involvement of neurological symptoms in response to the exposure to low quality outside and indoor air quality, bearing sub-toxicological levels of VOCs and other environmental chemicals able to trigger sick building(home) syndrome and MCS abnormal reactions [17,20]. Early neuro-physiological studies in the nineties have raised the hope to detect a specific abnormal functional brain reaction to low dose airborne chemicals or a neurotoxic damage in the brain of MCS patients by position emission tomography (PET) [137] or single photon emission computer tomography (SPECT) [138], thereafter often applied in the clinical practise to MCS patients for selective diagnosis and legal compensation purposes. Various neurological investigations on limited numbers of MCS or CFS subjects with both PET and SPECT have shown limited significant functional limbic or cortical alterations, in response to challenge with volatile chemicals. Nevertheless, overall results have failed so far proving with sufficient specificity the occurrence of neurotoxic or neuro-immunologic changes *vs.* control subjects [139,140]. Possible detection of specific brain function alterations of significant relevance though imaging techniques should be better confirmed in larger standardised provocation studies with low-dose exposure, comparing chemically sensitive subjects *versus* normal subjects having a previous experience of chemical exposure without adverse effects in order to assess unequivocally relevance of results and to exclude confounding psychological factors.

## 4. State-of-the-Art of Treatments

Consequent to the limited knowledge of etio-pathogenetic mechanisms, and to the absence of recognized diagnostic criteria and validated biomarkers of disease, environmental physic-chemical hypersensitivities entirely lack clinical *consensus* regarding therapeutic guidelines. The most urging concern is the complete lack of targeted effective and safe drugs, encouraging an unregulated and wide array of experimental protocols, including environmental medicine techniques, holistic therapies, individualized antioxidants (principally glutathione) or immune-modulating nutritional supplements, detoxification techniques, *etc.* [1,2,17,141].

Almost all approaches lack a documented rationale validated *in vivo*, and have never been controlled for safety and efficacy alone or *vs.* placebo, in controlled clinical trials. Based on the limited information so far available in the literature, treatments are prescribed in an allegedly individualized fashion. Nevertheless, drugs and their administration schedules are often uncorrelated with effective biochemical *status* and/or genetic predisposition of the single patients, and not thoroughly followed-up for compliance, clinical outcomes, and adverse effects monitoring. As a result, the main and only treatment approach for MCS that is documented in terms of both clinical outcome assessment and patient satisfaction is trigger removal, or exposure discontinuation [141], while no significant beneficial effect has been so far rigorously reported for any pharmacological or nutriceutical protocol. Main treatment approaches reported in the scant peer-reviewed literature reports available are listed in Table 2.

In this review, we have reported the most relevant information supporting the hypothesized impairment of the chemical defensive system in the onset and symptom perpetuating of MCS and related syndromes. Chronic invalidating environmental SRI most probably bear both genetic and metabolic components. Up-to-date, specific polymorphisms have been identified in the genes encoding for phase I and phase II detoxifying and antioxidant enzymes, and for their receptors and transcription factors, modifying gene activity and regulatory properties, and possibly representing main determinants of individual metabolizing capability [18]. On the other hand, individual peculiarity of adaptive response to chemical stressors maybe determined at the epigenetic level through the direct modification of biologically relevant macromolecular targets controlling gene expression, or either at transcriptional level. Chronically persistent toxic compounds may in fact chemically modify proteins to form auto-antigens, as suspected on the basis of symptom overlapping with classical autoimmune diseases [18,142]. As previously discussed, notwithstanding the inconclusive data on the prevalence of specific gene polymorphisms in MCS or other related conditions, fast or slow metabolizers could encounter severe difficulties in detoxification of environmental-borne chemicals and common drugs, such as antibiotics and anesthetics. In addition, since most environmental intolerances, and in particular MCS, are commonly regarded as psychiatric or somatoform disorders [22], many patients worldwide are prescribed psycho-active or pain-killer drugs with antidepressant, anxiolytic, analgesic, myorelaxant actions, without any preventive pharmacogenetic screening. Data acquired on polymorphic genes for drug metabolizing enzymes, affecting the metabolic rate for a number of psychotropic medications [99,143], raise a strong warning on the risk of severe adverse reaction in metabolically impaired patients. Of note, to our knowledge no clinical studies have so far monitored the frequency of adverse reaction to psychotropic drugs in subjects complying with diagnostic criteria of any of the various SRI. These reactions may be of frequent occurrence, as suggested in a study on patients’ self-reported reactions to treatments in MCS [141]. Evidently, the difficulties connected with extended genetic screening of population at risk are related to costs and interpretation, and additionally more research is needed to design innovative drugs delivering appropriate metabolizing enzymes to genetically impaired subjects. At present, following pharmacogenetic screening, common management of drug intolerance, including discontinuation of the offending medication, remains the first aid [144].

The best documented example of the beneficial clinical effects of trigger/incitant removal as a therapeutic approach is dental amalgam. Removal of amalgam fillings has been proven to resolve oral mucosa granulomatosis or lichenoid reactions attributed to Hg sensitization [145,146]. Removal significantly decreased blood titres of anti-thyroid autoantibodies *vs.* untreated controls, in autoimmune thyroiditis patients with Hg sensitization, while amalgam removal showed no immunologic effect in non-Hg sensitized patients [123]. The long persistence of mercury in human tissues [147,148] poses in any case a difficult problem, continuing after dental filling removal, due to the uncertain efficacy and possible counter-active effects especially towards the nervous system, of both synthetic and natural chelators, like glutathione, N-acetylcysteine, vitamin C, alpha-lipoic acid, *etc.*, administered for Hg removal, especially from the nervous system [71,149,150]. The possible beneficial effects towards the toxicity of environmental mercury, reported for selenium and vitamin E integrative protocols [151], encourages intervention studies on the largest array of environmental intolerance-related disorders, where the general relevance of nutritional and nutrigenomic approaches is still largely under-evaluated.

## 5. Conclusions: Sanitary System and Public Social Awareness

Notwithstanding the lack of consensus in the clinical community, the accumulating epidemiological evidences, and the compelling instances of patient organizations have brought single countries to recognize at least in part the organic pathological *status* of main SRI. As leading examples, in Europe notably Germany and Austria have listed MCS, FM and CFS under the ICD (International Classification of Diseases)-10 code of the World Health Organization with local modifications [1]. The same occurred in Japan in 2009, where special attention is given to indoor pollution, causing SBS/SHS [152], considered a point of attention also by Canada [153]. Sweden has recently recognized electro-magnetic hypersensitivity as a functional syndrome [142], whereas United States and Australia do not list any form of environmental sensitivity to ICD-10 as yet, though several leading state agencies and medical associations have since long recognized chemical hypersensitivity as a disability deserving deeper investigation [20,154].

Available data provide indirect evidence, awaiting further confirmation, that functional or/and genetic defects of endogenous enzymes detoxifying peroxides or stable toxic products of lipid oxidation like 4-HNE, may cause chronic oxidative stress and consequent metabolic alterations characteristic for the patients with MCS and other multi-organ pathologies connected with environmental intolerances. Sustained organic damage occurring from chronic exposure to ambient doses of environmental toxicants, presumably involves specific malfunction of detoxification pathways, so far still unidentified, displaying very high affinity for low-dose substrates [155]. More systematic studies are needed on the regulation, expression, induction, and activity of GPxs, peroxiredoxins, catalase and GST, to better understand pathological susceptibility to low-level external stimuli in these peculiar clinical settings, and therefore validate conclusively possible related biomarkers as essential tools for the correct processes of diagnosis, prognosis and treatment follow-up processes. Today, the only therapeutic approach with demonstrated beneficial effects is exposure avoidance, with the consequent complex task of removing putative indoor triggers from the living environment, often impossible in working and public places. Based on present available information, selected therapeutic proposals based on active antioxidant/chelator/gene- and immune-modulating principles, able to selectively prevent formation and release of excess reactive species or hydro- or lipid-peroxides, and to enhance specific detoxification pathways through ROS/RNS modulation, are to be taken into consideration for future clinical trials, which will be possible only if and when appropriate medical infrastructures with specific environmental requirements will be provided for these patients on the large scale [156].

In conclusion, it must be highlighted that a key difficulty for clinical research progress in this area lies in establishing dedicated sanitary structures for SRI patients, that should involve a complex network of expertises able to guarantee a satisfactory interdisciplinary clinical approach to diagnosis and treatment. Setting up these facilities and clinical approaches would also prove beneficial for the larger portion of patients suffering from allergies or other conventional diseases or conditions causally implicating environmental or professional factors. The concern about increasing health problems connected with environmental hypersensitivities is rather old-dated, as is the theoretical consensus on the need to provide appropriate infrastructures free of chemical barriers for disparate activities: patient consulting, hospitalization and surgery [157,158], dentistry, pharmacogenomic diagnostics to prevent iatrogenic adverse effects especially connected with antidepressants, analgesics, anesthetics, antibiotics [17,159], specialized training of sanitary personnel [160], and so on. In addition, adequate environmental units should be created, to perform provocation studies and other specific research in the absence of bias caused by background chemical or electro-magnetic interference [161]. Notwithstanding all recommendations and existing studies, accounting for possible sparing of health assistance costs through the adoption of a sanitary policy for MCS [162,163], the general commitment of healthcare systems around the world is so far negligible. Both sanitary and social system awareness should be supported by convincing epidemiological data on the effective prevalence of these disorders in the common population, and on a reliable assessment of the risk for the healthy predisposed subject of getting the disease in the near future.

No matter how uncertain case definitions of SRI still are, there remains the widely spread medical concern for the lower levels in quality of life of these patients, as compared to healthy individuals [1,164,165].

## Figures and Tables

**Table 1:** Main gene variants studied in MCS groups.

gene variant	population	diagnostic criteria	patients	healthy controls	Ref.
PON1 Q/R	GWV	-	25	20	Haley *et al.* [109]
PON1-55, PON1-192, PON2-148, CYP2D6, NAT1,NAT2, MHTFR C677T	MCS women	University of Toronto Health Survey (UTHS) reproducible, self-administered questionnaire	203	162	McKeown-Eyssen *et al.* [98]
NAT2, GSTM1, GSTT1	Chemically sensitive	Modified EESI questionnaire	273	248	Schnackenberg *et al.* [99]
5HTT, NAT1, NAT2, PON1, PON2, SOD2	Self-reported MCS	Self-administered Environmental Medicine Questionnaire questionnaire (Hüppe *et al.* [110])	59	40	Wiesműller *et al.* [67]
UGTA1	MCS and/or FCS and/or FM		42	-	Műller *et al.* [111]
CYP2C9, CYP2C19, CYP2D6, CYP3A5, UGTA1, UGTA, GSTT1, GSTM1, GSTP1	MCS	Cullen’s criteria, QEESI	144	218	De Luca *et al.* [17]
CYP2D6, NAT2, PON1, MHTFR, CCK2R	MCS	Cullen’s criteria	96	1,027	Berg *et al.* [112]

MCS = multiple chemical sensitivity; CFS = chronic fatigue syndrome; FM = fibromyalgia; GWV = Gulf War Veterans.

**Table 2:** Main categories of treatments (including non-conventional and self-prescription) reported for environmental intolerances.

Treatment	Condition
Trigger removal *(trigger-free living space, chemical avoidance, antifungal sanitization, working place change or abandon, selected food removal, dental amalgam removal*	MCS, CFS, IBS, EHS, SBS, SHS
Nutritional supplements	MCS, FM, FCS
Prescription therapies (*psychotropic drugs, antimycotics, antiobiotics, hormone replacement)*	MCS, FM, FCS, IBS, GWV
Psychotherapy	MCS, FM, CFS, GWV, dental amalgam disease, EHS
Detoxification treatments	MCS, FM, CFS, GWV, dental amalgam disease
Holistic treatments	MCS, FM, CFS, GWV, dental amalgam disease
Body therapies	MCS, FM, FCS

MCS = multiple chemical sensitivity; CFS = chronic fatigue syndrome; FM = fibromyalgia; IBS = irritable bowel syndrome; SBS/SHS = sick building/house syndrome; GWV = Gulf War Veterans.
